# Time-dependent X-ray diffraction studies on urea/hen egg white lysozyme complexes reveal structural changes that indicate onset of denaturation

**DOI:** 10.1038/srep32277

**Published:** 2016-08-30

**Authors:** Tushar Raskar, Sagar Khavnekar, Madhusoodan Hosur

**Affiliations:** 1 Tata Memorial Centre/Advanced Centre for Treatment Research and Education in Cancer, Kharghar, Navi Mumbai, 410210, India

## Abstract

Temporal binding of urea to lysozyme was examined using X-ray diffraction of single crystals of urea/lysozyme complexes prepared by soaking native lysozyme crystals in solutions containing 9 M urea. Four different soak times of 2, 4, 7 and 10 hours were used. The five crystal structures (including the native lysozyme), refined to 1.6 Å resolution, reveal that as the soaking time increased, more and more first-shell water molecules are replaced by urea. The number of hydrogen bonds between urea and the protein is similar to that between protein and water molecules replaced by urea. However, the number of van der Waals contacts to protein from urea is almost double that between the protein and the replaced water. The hydrogen bonding and van der Waals interactions are initially greater with the backbone and later with side chains of charged residues. Urea altered the water-water hydrogen bond network both by replacing water solvating hydrophobic residues and by shortening the first-shell intra-water hydrogen bonds by 0.2 Å. These interaction data suggest that urea uses both ‘direct’ and ‘indirect’ mechanisms to unfold lysozyme. Specific structural changes constitute the first steps in lysozyme unfolding by urea.

More than twenty human degenerative diseases^1^,^2^,^3^ are caused by aggregation of misfolded proteins into amyloid fibrils. Similar misfolding and amyloid-like fibril formation occurs with soluble wild-type hen egg-white lysozyme (HEWL) under certain conditions^4^. Therefore, understanding the pathways of protein unfolding using HEWL may help develop treatments for such degenerative diseases. Urea is one of the small molecules known to unfold proteins, but the exact mechanism of unfolding is not understood despite a variety of theoretical and experimental studies^5^,^6^,^7^,^8^,^9^,^10^,^11^,^12^,^13^,^14^,^15^,^16^,^17^,^18^,^19^,^20^,^21^. Urea-denaturation curves can be explained equally well by two different thermodynamic models: the solvent-exchange model and the site-binding model^22^,^23^,^24^,^25^. These models form the basis of two different denaturation mechanisms: an “indirect mechanism”^6^,^26^,^27^,^28^,^29^ in which urea affects the hydrogen bonding network of solvent water in such a way that the hydrophobic effect is neutralised and the unfolded state of the protein becomes energetically favourable over the folded state; and a “direct mechanism”^30^,^31^,^32^ that assumes stronger direct binding of urea over water. Furthermore, the nature of urea binding to folded and unfolded proteins is thought to be similar, with urea facilitating denaturation simply because a larger number of binding sites are exposed in the denatured state^33^. This mechanism does not explain how the kinetic barrier to unfolding is overcome. One possibility could be that the unfolded conformation is spontaneously formed^34^. The other possibility is that because of chemical similarity to the peptide group, urea competes with native interactions thereby destabilising the folded state of the protein^26^. The relative importance of different types of chemical interactions between urea and the protein is the major point of discussion in the direct mechanism^35^,^36^,^37^,^38^,^39^,^40^. A two-stage direct denaturation mechanism has been proposed based on micro-second Molecular Dynamics (MD) simulations on lysozyme in 8 M urea. The driving force in the first stage of this mechanism is the favourable protein-urea van der Waals interactions compared to protein-water interactions^35^. Although kinetic evidence in support of this two stage mechanism is recently reported^11^, experimental information on structural aspects of urea binding to proteins is limited. Here, we present high-resolution crystal structures that map urea binding to lysozyme as a function of time. These results should help in evaluating MD simulation data on protein unfolding by urea^11^,^41^. Crystals of the complexes were prepared by soaking native crystals in freshly prepared buffer solutions containing 9 M urea for different periods of time. The complex structures reveal good electron density for a total of forty-seven urea molecules occupying twenty-one different positions replacing forty-nine water molecules in the first solvent shell. The urea molecules form far more van der Waals interactions with the protein compared to displaced water molecules, in agreement with results of MD simulations^35^. We also find that urea affects first-shell water-water hydrogen bond network. Thus our data suggest that protein denaturation by urea is through both ‘direct’ and ‘indirect’ mechanisms. Urea-binding has led to the loss of a few intra-protein hydrogen bonds, and this, we propose, represents the start of the pathway of lysozyme denaturation by urea.

## Results

### Exponential decrease in diffraction

Table 1 gives crystal data and refinement statistics for the five structures identified as ‘native’, ‘2 h’, ‘4 h’, ‘7 h’, and ‘10 h’. These structures are of HEWL crystals soaked in 9 M urea solutions at pH 3.5 for 0, 2, 4, 7, and 10 hours, respectively. The five crystals are isomorphous, belong to the tetragonal space group P4_3_2_1_2, and diffract X-rays to a resolution of approximately 1.6 Å. Native crystals soaked for twelve and a half hours had almost three fold higher mosaicity compared to 10 h, and diffracted to much lower resolution compared to the other five structures (Data not shown). When soaked for longer than thirteen hours, native crystals completely dissolved. These observations indicate that the crystal disorder increases exponentially within a short period beyond the soak time of 10 hours, as reported previously^19^. This situation is akin to the sigmoidal transition observed in unfolding of proteins in solution due to co-operativity in urea-binding^42^. The average B-factors for the protein, the urea molecules and the water molecules are significantly higher in 10 h compared to others (Table 1), consistent with a possible onset of denaturation in 10 h. The radius of gyration of the protein molecule in all structures is almost identical.

### Urea replaces water in the first solvent shell

**Figure 1:**
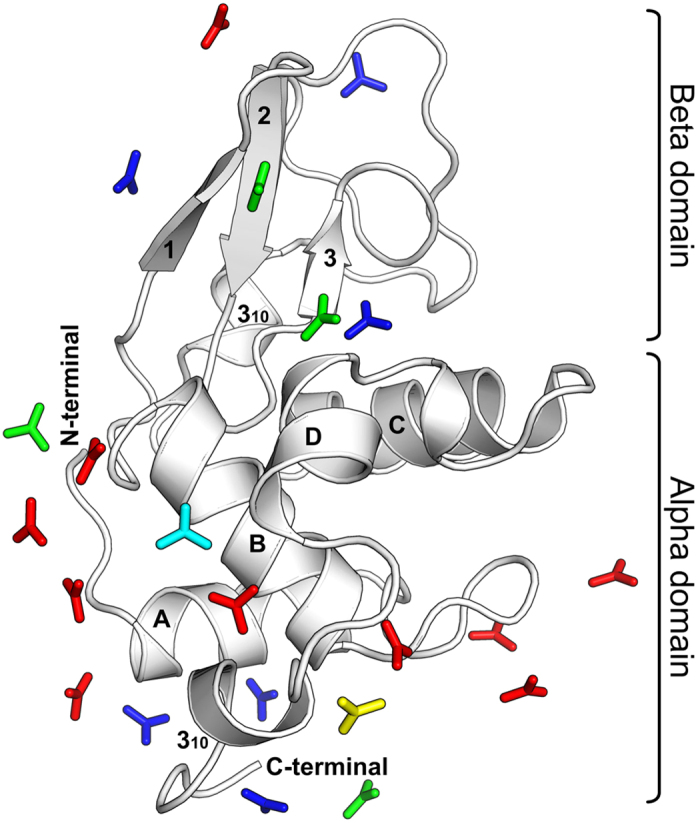
Urea molecules bind in specific pockets on the lysozyme surface. Urea molecules are shown in stick representation while protein is shown as a cartoon. The secondary structure elements are labeled. All urea molecules are colour coded: Blue (present in 2 h, 4 h, 7 h and 10 h), Yellow (present in only 4 h), Cyan (present in 2 h, 7 h and 10 h; but not in 4 h), Green (present in 7 h, and 10 h), and Red (present in 10 h).

The full length HEWL comprises 129 amino acid residues arranged in two domains: an α-domain containing four α–helices (A, B, C and D) and two 3_10_ helices, and a β-domain containing one three-stranded β-sheet [strand 1 (residues 43–46), strand 2 (residues 51–54), and strand 3 (residues 58–60)] and a long loop region (Fig. 1). There are four disulfide bonds in the molecule. Figure 1 also shows, in stick representation, positions of the twenty-one urea molecules bound to lysozyme in the crystal. It is clear that all urea molecules are bound on the protein surface, with a majority of them binding to the α-domain. Two molecules are bound in the active site cleft between the two domains. Positions observed after shorter soak times are a subset of positions observed after longer soak times, except for the soak time of four hours. Though seven urea molecules are bound both in 2 h and 4 h, one urea molecule differed in its position. The number bound in 7 h is twelve, which includes the seven in 2 h, while the twenty-one bound in 10 h include the twelve bound in 7 h. The twenty-one urea molecules are not segregated, as only two of them have any interactions with one another^12^,^29^. Only four of the twenty-one urea molecules bind to two symmetry related protein molecules in the crystal. The urea molecules form hydrogen bonds to both water and protein (Tables 2 and 3). About 71% urea molecules form direct hydrogen bonds to protein atoms, with 41.6% to 47.6% forming multiple direct hydrogen bonds. The number of urea-protein hydrogen bonds per urea molecule is approximately 2.5 for all soak times, while the corresponding number for urea-water hydrogen bonds is 1.5. On the other hand, the average numbers of water–protein and water-water hydrogen bonds per water molecule are similar at 1.7 and 1.5, respectively. Thus, compared with water, urea shows a higher propensity to interact with protein through hydrogen bonding. In a majority of the hydrogen bonds to backbone, urea is a donor, while it is an acceptor in a majority of the hydrogen bonds to side chain atoms. Urea molecules interact directly with protein atoms also through their pi-electron cloud (Supplementary Figure S1). Preference for urea is also observed in van der Waals interactions from protein atoms. The number of van der Waals interactions with protein is higher for urea compared to those for displaced water molecules, by almost 100% in 2 h, 4 h, 7 h and 10 h (Table 3).

### Urea binds first to the lysozyme backbone

The urea-protein interactions involve both the backbone and side chain atoms of the protein. The percentages of hydrogen bonds to the backbone are 67%, 75%, 57% and 44% respectively, in the 2 h, 4 h, 7 h, and 10 h structures (Table 2). The additional five urea molecules at 7 h compared to 2 h form fifteen hydrogen bonds, of which five are to the backbone atoms, giving the percentage of backbone binding as 33%, which is much lower than 67% seen in 2 h. Similarly, only 28% of the hydrogen bonds by the nine additional urea molecules at 10 h compared to 7 h are to the backbone atoms. While the percentages of urea-backbone hydrogen bonds decrease with increase in soak time, the number of hydrogen bonds to side chain atoms increases, resulting in an increase in the total number of urea-protein hydrogen bonds from nineteen in 2 h to fifty-two in 10 h (Table 2). As the soaking time is increased, the number of protein-urea van der Waals contacts increases from 278 to 600, while protein-water contacts decrease from 1156 to 1028 (Table 3). The seven urea molecules in 2 h make 155 contacts to backbone atoms, giving an average of twenty-two contacts per urea molecule. The five additional urea molecules in 7 h form 156 extra contacts over 2 h and, of these, thirty-five are to the backbone atoms, giving an average of only seven contacts per urea molecule. Similarly, between 7 h and 10 h, each of the nine additional urea molecules makes on an average, seven contacts to the backbone atoms. Thus, hydrogen-bonding and van der Waals interactions of urea with folded lysozyme are first to the lysozyme backbone and only later to side chain atoms. By contrast, the percentage of water- protein hydrogen bonds to the backbone remains unchanged at 54%, 54%, 52% and 52% for the four soak times. Similarly, the percentage of water-backbone van der Waals interactions also remains unchanged (49% to 52%, Table 3) for the four soak times. The average lengths of hydrogen bonds from urea to backbone amide and carbonyl groups are 2.9 Å and 3.0 Å, while the corresponding values for water are 3.1 Å and 2.8 Å, respectively. It thus appears that urea forms stronger hydrogen bonds to the backbone amide while water forms stronger hydrogen bonds to backbone carbonyl groups. Interestingly, the majority of urea-backbone interactions do not involve residues that are part of the secondary structure of lysozyme.

### Urea shows only marginal preference for polar residues

The ratio (urea-protein van der Waals contacts to polar side chains)/(urea-protein van der Waals contacts to apolar side chains) has a value of approximately 1.1 for all soak times, showing a marginal preference to interact with polar residues. The corresponding ratio for water molecules increases from 1.5 in the native structure to around 1.7 in all urea-soaked structures, suggesting that the presence of urea promotes non-bonded interaction of water with polar residues, in agreement with theoretical predictions^41^. When urea-protein hydrogen bonds were analysed, it was found that the number of hydrogen bonds to polar charged side chains is higher than that to polar uncharged side chains. The solvent accessible surface areas of the protein atoms in the five structures only marginally differ from one another (data not shown). However, the backbone is systematically more accessible in the urea complexes compared to the native structure. Also, polar side chains are more accessible, suggesting that urea binding results in exposure of backbone and polar atoms.

### Urea solvates hydrophobic residues

**Figure 2:**
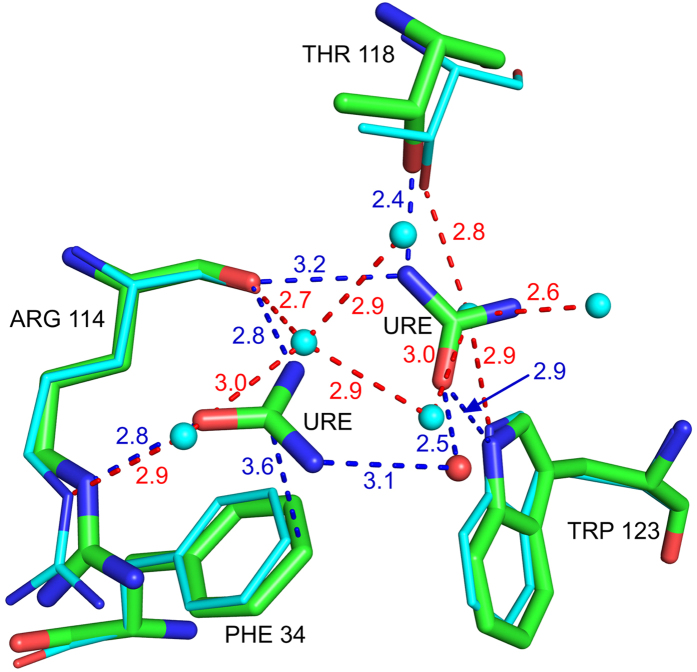
In the urea complex (green) urea solvates hydrophobic residues Phe 34 and Trp 123 by replacing five tetrahedrally hydrogen bonded water molecules (cyan).

As the number of urea molecules bound to the protein increases from seven in 2 h to twenty-one in 10 h, the number of first-shell water molecules progressively decreases from 155 in native to eighty-two in 10 h. Sixteen to eighteen of these water molecules are from the inter-domain interface, and they shield exposed hydrophobic residues like Ala 107, Ile 98, Trp 63, Ile 58 and Val 109. Many of these water molecules are replaced by the two urea molecules bound in the active site cavity (Fig. 1). In the native structure, six water molecules form a tetrahedrally hydrogen bonded cage around the hydrophobic residues Phe 34 and Trp 123. In the urea-soaked structures, all these water molecules are replaced by the combination of one water in a newer position, and two urea molecules hydrogen bonding directly with Thr 118 OG, Arg 114 NH and Trp 123 NE1 (Fig. 2).

### Urea alters the water-water hydrogen bonding network

Although there is an enrichment of urea molecules in the first solvent shell, there are still many water molecules in the first solvent shell of the protein. There are many hydrogen bonds among these water molecules. The average number of hydrogen bonds per water molecule decreases from 1.0 in the native structure to about 0.7 in 10 h (Supplementary Table S1). The average length of the water-water hydrogen bonds is also altered in urea-soaked structures. It progressively decreases as the soak time increases, ultimately reaching the value 2.75 Å in 10 h. This length is significantly shorter compared to the average length of 2.95 Å in the native structure. Since hydrogen atoms are not visualised, we have not analysed variations in the hydrogen bond angle. These observations suggest that urea brings about a change in water -water interactions and dynamics.

### Urea binding causes changes in protein structure and interactions

**Figure 3:**
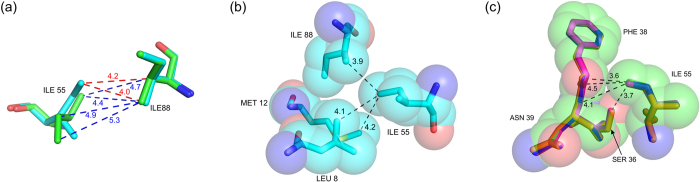
Urea-binding alters Ile 55 rotamer and its interactions: (**a**) contacts with Ile 88 in 10 h (green) and native crystals (cyan), (**b**) space filling model showing aliphatic environment of Ile 55 CD1 in native crystals, (**c**) space filling model showing partly polar environment around Ile 55 CD1 in 2 h, 4 h, 7 h, 10 h soaked crystals.

**Figure 4:**
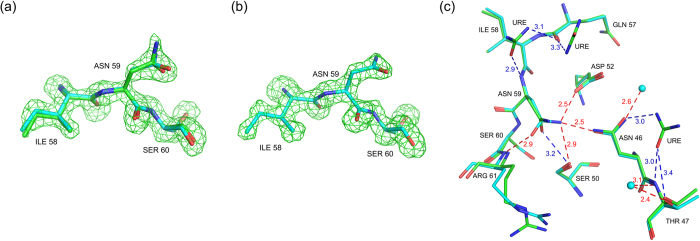
Simulated annealing (mF_o_–DF_c_) omit maps for Ile 58, Asn 59, and Ser 60 residues. The maps are contoured at 2.8 σ: (**a**) The 10 h (green) structure with superposition of the native structure (cyan) (**b**) native structure, and (**c**) hydrogen bonds from urea to Thr 47 and Asn 46 that cause a flip in Asn 46 and Asn 59 rotamer change in 10 h. Water molecules hydrogen bonding to Thr 47 and Asn 46 in the native structure are also shown. The hydrogen bond lengths are labeled: native (red), 10 h (blue).

**Figure 5:**
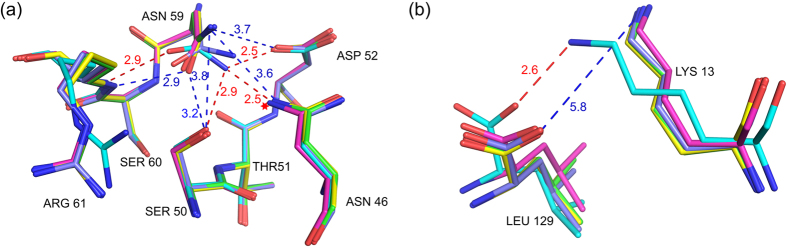
Structural alterations due to urea-binding: (**a**) rotamer change in Asn 59 and loss of side-chain hydrogen bonds in 2 h, 4 h, 7 h, and 10 h, (**b**) loss of ionic hydrogen bond between Lys 13 NZ and 129 COO^−^ in 2 h, 4 h, 7 h and 10 h. Hydrogen bond distances are labeled: native (red), 10 h (blue).

The overall conformation of lysozyme is similar in the five structures with Cα RMSD’s in the range 0.2 Å–0.3 Å. However, there are significant changes in the B-factors and positional parameters of specific residues (Supplementary Figure S2). The average protein B-factor first decreases before starting on the upward trend, suggesting that urea first stabilizes the protein conformation before unfolding it, as has been observed by others^26^. There are three types of structural changes due to urea binding: (1) positional shift in protein main chain, (2) rotamer change in amino acid residues, and (3) shift in position of buried water molecules. Residues in the β-domain loops (40–79), and in helices A (5–14) and D (109–114) show significant shifts. For example, in 10 h, the Cα atoms of residues 13, 14, 15, 109 and 110 shift by 0.79, 0.87, 0.66, 0.94 and 0.80 Å respectively, while the terminal carboxyl C moves by 1.6 Å, when compared to the native structure. As a result, the distance Lys 13 Cα - Leu 129 Cα, changes from 7.94 Å in native to 6.71 Å in 10 h. The Ile 55 Cα in 10 h is displaced by 0.4 Å from its native position. This shift is caused by the hydrogen bond between Leu 56 CO and Trp 108 NE1, which itself shifts because of urea - Trp 108 CO hydrogen bond. Rotamers of several residues including Lys 13, Arg 14, Asp 18, Asn 19, Ile 55 and Asn 59 change on urea-binding. Rotamer changes in Ile 55 and Asn 59 are particularly significant. The χ1 dihedral angle of the core residue Ile 55 becomes −173° in 10 h from 54.5° in the native structure (Fig. 3a). This alteration is to avoid short contacts with Ile 88 CG2 (3.17 Å) and Thr 40 CG2 (3.06 Å) that would result if the native rotamer was retained in the shifted position of Ile 55 Cα. The native and changed contacts from Ile 55 CD1 are shown in Fig. 3b,c respectively. The χ2 dihedral angle of Asn 59 changes substantially as confirmed by the simulated annealed omit maps calculated by omitting residues 58–60 (Fig. 4a,b). This altered conformation is to avoid steric and charge repulsion with Asn 46 ND2 (2.4 Å), which itself undergoes a flip in the χ2 angle because of hydrogen bonding interactions from urea to Asn 46 OD1, Thr 47 NH, and Thr 47 OG (Fig. 4c). Consequently, side-chain hydrogen bonds involving the highly conserved residues Asn 59, Asp 52, Asn 46 and Ser 50 are lost in 2 h, 4 h, 7 h and 10 h structures (Fig. 5a). The conformation of Lys 13 is also altered in the four urea complexes reported here. Hydrogen bonding from urea molecules to Arg 14 NH2 and 129 COO^−^, and to CO and NH of Arg 128, causes the C-terminus to shift by 1.6 Å. The combined effect of these two changes is the loss of ionic hydrogen bond between Lys 13 NZ and Leu 129 COO^−^ holding together the two ends of the native protein (Fig. 5b). As the third structural effect of urea-binding, there is a shift, by 1.04 Å, in the position of one of the four buried water molecules. Since these water molecules are conserved, their positions may be important for the stability of lysozyme^43^.

### Restrained dynamics

**Figure 6:**
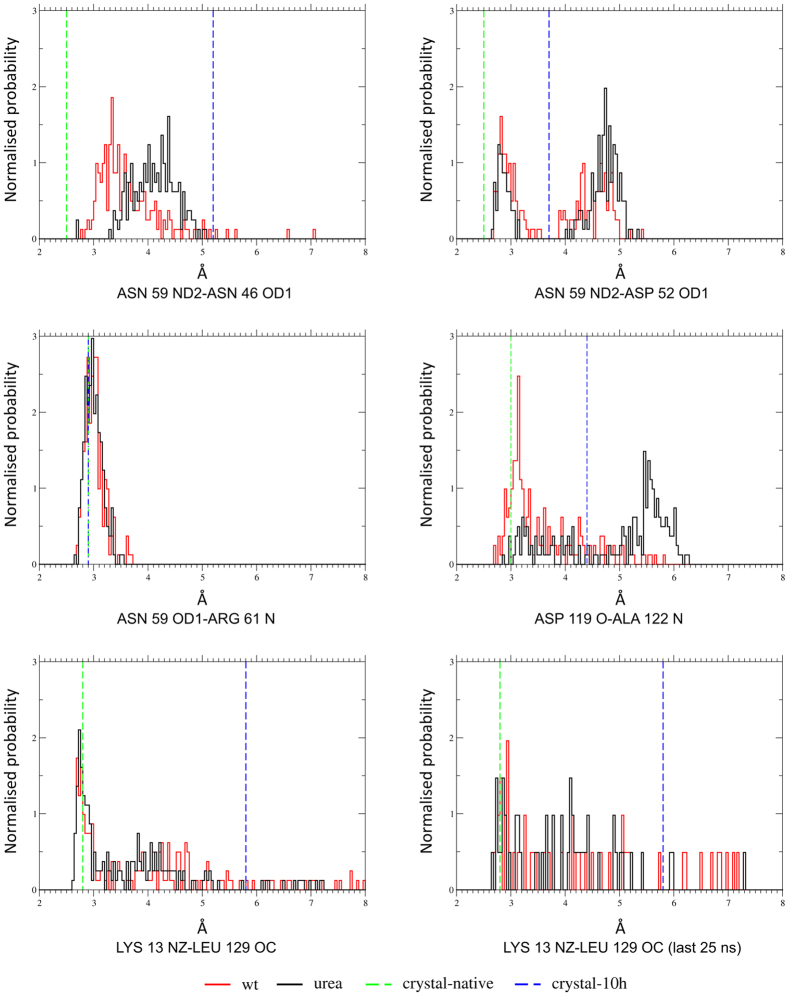
Distance histograms for the five hydrogen bonds affected in 10 h. Control simulation (red), transition simulation (black). Crystallographic distances for native (green) and 10 h (blue) are shown as vertical dashed lines.

Molecular Dynamics simulations were performed to explore conformational transition and local dynamics in native HEWL when bound by urea molecules as observed in 10 h. Positions of twenty one urea molecules as determined in 10 h combined with native lysozyme coordinates (in the absence of urea) were used as the starting model in the simulation. Urea molecules were restrained in order to explore local changes in the protein due to urea molecules bound at these sites. This simulation will be referred to as ‘transition simulation’. Simulation performed with the native lysozyme structure and water as a solvent was treated as the control. The results over the course of molecular dynamics simulation for 100 ns are shown in Fig. 6, as distance histograms, which show donor - acceptor distances for the five selected hydrogen bonds. Shift of peak in the distance distribution from 3.3 Å in the ‘control simulation’ to 4.5 Å in the ‘transition simulation’ for the Asn 59 ND2 - Asn 46 OD1 hydrogen bond clearly indicates breakage of this hydrogen bond. Similarly, we observed a peak shift from 3.2 Å to 5 Å indicating breakage of the Asp 119 O - Ala 122 N hydrogen bond. In the case of Asn 59 ND2 - Asp 52 OD1 hydrogen bond, the major peak in the distance distribution is around 2.8 Å for the control simulation, whereas it is around 4.8 Å for the transition simulation, indicating breakage of this hydrogen bond. For the Asn 59 OD1 - Arg 61 N hydrogen bond, no change is observed between the two simulations, again in agreement with our crystallographic findings. Though distance distribution for Lys 13 NZ - Leu 129 O hydrogen bond is similar initially for both the control and transition simulations, distribution calculated over the last 25 ns showed a shift from 2.8 Å to 4.2 Å in the transition simulation, suggesting that this hydrogen bond may break later than the ones listed above.

## Discussion

### Urea protein interactions

Urea binding to proteins in solution has been probed in a variety of thermodynamic and spectroscopic experiments^24^,^28^,^33^. These studies show that interactions of nonpolar groups with urea are enthalpically unfavorable but entropically favorable, while urea-polar group interactions are enthalpically favorable but entropically unfavorable. Empirical data shows that the conformational stability of the protein is linearly dependent on the concentration of urea present^24^. The slope of this linear dependence, denoted as *m*, is highly correlated to the changes in accessible surface area, especially of backbone atoms^20^. High concentrations (8 M–10 M) of urea are needed for complete denaturation, indicating that urea-protein interactions are weak and non-specific^34^. However, some NMR and hydrogen exchange experiments provide evidence for site-specific binding^44^,^45^,^46^,^47^,^48^. These solution studies do not describe the detailed geometry of urea-protein interactions, which can be obtained accurately by crystallography. Urea binding to proteins has been investigated using crystallography^14^,^15^,^16^,^17^,^18^,^19^,^49^, but probing binding as a function of time has not been reported hitherto. In earlier studies of urea binding to lysozyme^14^,^15^, three binding sites were observed in the triclinic crystals as against nine in the tetragonal crystals. All these nine sites are found in the present study. Our work has revealed twelve additional urea-binding sites possibly because of longer soak times, more complete and better data quality and improved refinement procedures. The maximum number of twenty-one urea binding sites observed in 10 h are still only 18% of the 119 sites estimated earlier through calorimetric studies^50^. It is possible that the remaining sites would be occupied for soaking times longer than 10 hours, but we are unable to study those crystals because of rapid loss in diffraction due to increased crystal disorder. The occupancy of urea molecules in these twenty-one sites is either 0% or 100% depending on the soak time. For example, in 10 h the occupancy is almost 100% for all sites. However in 2 h, 4 h, and 7 h, the occupancy is 100% only for seven (2 h), seven (4 h) and 12 (7 h) of these twenty-one sites, and is 0% for the remaining sites. This observation is in sharp contrast to the suggestion that urea molecules occupy preferred binding sites in BPTI and PEC-60 for less than 10% of the times even in 8 M urea^34^. The average number of water molecules replaced per urea molecule is almost the same for all soak times with an overall average of 2.75, which compares well with the predicted value of 3^41^ and the measured value of 2.45^51^. There is a decrease in the number of protein solvent (urea and/or water) hydrogen bonds in urea-soaked structures compared to that in the native structure (Table 2). This result is in contrast to constancy of protein-solvent hydrogen bonds found in MD simulations on barnase^41^. Urea molecules bind both to water molecules and to specific protein sites through multiple hydrogen bonds, as also suggested by the calorimetric study of urea binding to lysozyme^50^. The fraction of urea molecules forming multiple hydrogen bonds to protein is almost three times that predicted by MD simulations on folded barnase^41^. The ratio of urea-backbone to urea-side chain hydrogen bonds decreases from 2.1 in 2 h to 0.8 in 10 h. Most of the binding sites are not from peptide groups forming secondary structure, but are from peptide groups in the loop regions of the tertiary structure, in agreement with predictions by MD simulations on barnase. Among urea-side chain hydrogen bonds, more charged residues are involved in 10 h than in 2 h structures. When van der Waals contacts to protein atoms are considered, the numbers are consistently higher for urea compared to those for displaced water molecules. The increase in the number of van der Waals contacts is 130, 131, 225 and 258 respectively in 2 h, 4 h, 7 h, and 10 h structures. These data clearly show that van der Waals interactions are the driving force for urea replacing water molecules in the first solvent shell of the protein^35^,^52^. Urea molecules show only a marginal preference for interaction with polar residues, in contrast to the six fold preference predicted by MD simulations on CI2^10^. One of the twenty-one urea molecules is doubly hydrogen bonded to a water molecule as anticipated through infrared spectroscopic studies^8^.

### Mechanism of denaturation

Two types of mechanisms, ‘direct’ and ‘indirect’, have been proposed to account for the observed denaturation curves^53^. In the indirect mechanism urea causes a change in the water-water hydrogen bond network around hydrophobic groups in proteins thereby increasing their solubility and weakening the hydrophobic effect^27^,^29^. NMR studies on the N-terminal domain of the repressor protein 434 in the presence of 7 M urea, also suggest solvation of hydrophobic residues by urea^54^. Though some experimental and theoretical results suggest that water-water interactions do not change to a great extent in the presence of urea^8^,^35^, the structural data presented here show that urea disrupts water-water hydrogen bond network around hydrophobic residues (Fig. 2). Furthermore, we find that presence of urea causes a decrease in the number of water-water hydrogen bonds, which provides evidence for the proposal that the water around urea is less hydrogen bonded than bulk water^12^,^27^,^40^. Our finding that urea causes shortening of the water-water hydrogen bonds is in agreement with the theoretical prediction of structural changes for the hydration shell water around carbonyl and amino groups of urea^27^,^28^. The stronger water-water hydrogen bond in the presence of urea can rationalise the greatly reduced diffusion and orientational flexibility of water molecules observed using 2DIR spectroscopy^8^,^13^,^26^.

In the direct mechanism, urea is proposed to interact directly with the protein to weaken intra-protein interactions. However, whether the direct interactions are with the hydrophobic side chains, the polar backbone, or both remains unresolved^35^,^53^,^55^. Our results show that urea binds directly to lysozyme through both hydrogen bonding and van der Waals interactions, initially to the backbone atoms and only later to the side chain atoms. Our experiments show that with increase in soaking time there is a systematic increase in accessible backbone surface area, which is shown to be correlated with the rate of change of the unfolding equilibrium^20^. Thus our results support the hypothesis that urea denatures proteins through both ‘direct’ and ‘indirect’ mechanisms, and that van der Waals interactions provide the driving force for direct binding. Our results further identify a few intra-protein hydrogen bonds that urea breaks, thereby playing an active role in unfolding lysozyme^26^.

### Onset of denaturation

**Figure 7:**
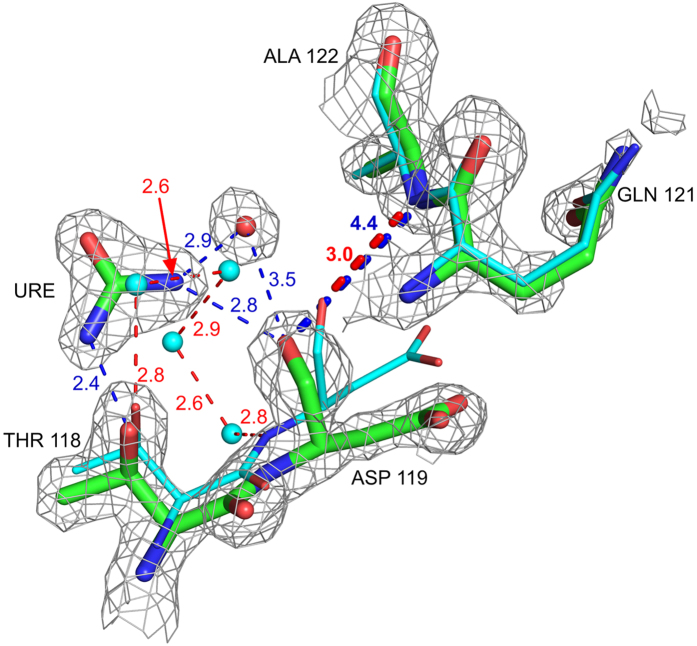
Urea binding to Thr 118 causes loss of 119 CO - 122 NH hydrogen bond. 3.0 Å in native (cyan) and 4.4 Å in the 10 h structure (green). Four water molecules from the native structure are also replaced by urea.

**Figure 8:**
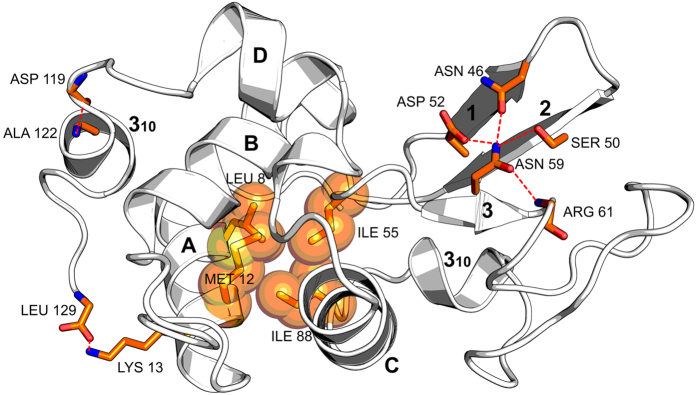
Cartoon representation showing alpha and beta domains in HEWL. Locations of suggested unfolding ‘hot-spots’ in the 3-D structure are shown in stick and space-filling models (orange).

The α and β domains of HEWL are linked by contacts between β-turn residues Ile 55 and Leu 56, and the hydrophobic patch in the α-domain. The alteration observed in the rotamer of Ile 55 in 2 h, 4 h, 7 h and 10 h structures is expected to disturb these contacts leading to destabilization and unfolding of the β domain^5^. The change in the rotamer of Asn 59 also will destabilize the β-sheet. In the native structure, there are three strong hydrogen bonds (Asn 59 ND2 – Asp 52 OD1 = 2.5 Å, Asn 59 ND2 – Asn 46 OD1 = 2.5 Å and Asn 59 ND2 – Ser 50 OG = 2.9 Å) between side chains of totally conserved residues from different strands of the three-stranded β-sheet. Because of the rotamer change, these hydrogen bonds are lost in 7 h and 10 h structures, leading to destabilization of the β-domain (Fig. 5a). In native lysozyme, the carboxy terminal COO^−^ and Lys 13 NZ form an ionic hydrogen bond (Fig. 5b), which is lost in 2 h, 4 h, 7 h and 10 h structures. This loss may destabilize HEWL, since the Lys13Ala mutant, in which this hydrogen bond is lost, possesses a T_m_ value 2.5 °C lower compared to native lysozyme^56^. The hydrogen bond between 119 CO and 122 NH is also lost following urea-binding (Fig. 7). This loss is because of hydrogen bonding of urea to Asp 119 CO and to Thr 118 O_γ_H. Overall, urea-binding affects stability of HEWL in multiple ways. Based on our observations, we suggest the following sequence for unfolding of HEWL by urea: (1) loss of Lys 13 NZ – Leu 129 COO^−^ hydrogen bond, (2) destabilization of β-domain through Ile 55 and Asn 59 rotamer changes, and (3) loss of hydrogen bond in the α-domain. The locations of these suggested unfolding ‘hot-spots’ in the 3-D structure of lysozyme are shown in Fig. 8. This sequence is consistent with earlier findings that urea destabilizes β-sheet structures first^26^,^57^.

## Conclusion

We provide here structural evidence for the first stage of the two-stage model for lysozyme denaturation previously proposed on the basis of microsecond Molecular Dynamics Simulations^35^. Using X-ray diffraction experiments, we mapped urea-protein interactions as a function of time. Twenty-one urea molecules bind to the protein surface in a phased manner and displace a total of forty-nine water molecules in the first solvent shell. The driving force for urea-binding in preference to water-binding is the increased number of urea-protein van der Waals interactions. Urea molecules bind first to the backbone and later to side chains of folded lysozyme. The binding leads to increased exposure of the protein backbone to the solvent. The binding also alters the solvent structure by disrupting the water-water hydrogen bond network around hydrophobic residues, and also by shortening water-water hydrogen bonds in the protein hydration shell. Urea-binding causes loss of: (1) three side chain hydrogen bonds from Asn 59 that stabilise the anti-parallel β-sheet, and (2) an ionic hydrogen bond between Lys 13 NZ and the carboxy terminus. There is also a change in the rotamer conformation of the totally buried Ile 55 residue, which contributes to the stability of the lysozyme fold. Our results give an atomic level picture of how urea initiates lysozyme unfolding by breaking hydrogen bonds first in the β-domain and later in the α-domain (residues 119–122).

## Materials and Methods

### Sample preparation

Hen egg white lysozyme obtained from M/S Sigma Aldrich Ltd., USA, was 99.9% pure, and was used without further purification. Single crystals were grown at 22° C by the vapour diffusion method in sitting drops of total volume 4 μL, obtained by mixing equal volumes of protein and precipitant solutions. The concentration of the protein was 25 mg/ml. The precipitant solution in the reservoir was 100 mM sodium citrate/HCl buffer of pH 3.5 containing 1.2 M sodium chloride. Tetragonal crystals appeared within a day, and were allowed to grow for a week before use. The hanging drop method was used to carry out the soaking experiments at 22 °C. Soaking solution was similar to the precipitant solution, but additionally contained 9 M urea. Six μL of the soaking solution were placed on a siliconised cover slip, and lysozyme crystals were transferred to this droplet using a cryoloop. This cover slip was then inverted and vacuum sealed over a reservoir containing 1.0 mL of soaking solution. The soaking experiments were carried out separately for soak periods of 2, 4, 7 and 10 hours.

### Data collection, data processing and model refinement

One soaked crystal was picked onto a cryoloop, dipped for about 20–30 seconds into the cryo-protectant (soaking solution containing 25% glycerol) and immediately mounted on the goniometer for data collection. X-ray diffraction data were collected, at liquid nitrogen temperature, by the oscillation method using the MARDTB system mounted on a NONIUS microstar copper rotating anode X-ray generator. The 1° oscillation frames were processed and scaled using MOSFLM, POINTLESS, and SCALA programs of CCP4 suite^58^. The structures were solved using the molecular replacement method as implemented in the software PHASER. Lysozyme structure from PDB with PDB ID 191L was used as the search model after removing ligands and water molecules. Crystallographic refinement used REFMAC5^59^ and PHENIX^60^ software packages. Simulated annealed omit maps were calculated using PHENIX. Computer graphics software COOT^61^ was used for the interpretation of (mFo–DFc) and (2mFo–DFc) electron density maps. The accessible surface areas and intra-protein hydrogen bond energies were calculated using the software package VADAR^62^. The AREAIMOL and NCONT sub-programs of CCP4 were used in the calculation of accessible surface areas and inter-atomic contacts respectively. The cut-off distances for hydrogen bond and van der Waals interaction calculations were 2.0 Å to 3.3 Å, and 3.3 Å to 4.7 Å, respectively. The first shell water molecules were identified using the PHENIX software package.

### Restrained dynamics

Restrained Molecular Dynamics (RMD) simulations were carried out using GROMACS^63^ software package, Amber 03 force field^64^, and TIP3P water model. The following two molecular systems were subjected to simulation: (1) native crystal structure of lysozyme in complex with twenty-one urea molecules from 10 h (transition simulation) and (2) native lysozyme in water (control). The molecular models were solvated in bulk water containing 150 mM KCl. Urea molecules were position-restrained in all three dimensions. Simulation systems were energy minimized using the method of steepest descent minimization, and then subjected to 1 ns dynamics under NVT and NPT. Production MD was then performed for 100 ns. Root mean square fluctuations were calculated from 100 ns trajectory for simulations 1 and 2 above. Intra-protein and protein-urea hydrogen bonds having occupancy greater than 25% were monitored. Hydrogen bond cutoff distance of 3.5 Å was used. The five hydrogen bonds which were affected in urea-soaked crystal structures were monitored.

## Additional Information

**How to cite this article**: Raskar, T. *et al.* Time-dependent X-ray diffraction studies on urea/hen egg white lysozyme complexes reveal structural changes that indicate onset of denaturation. *Sci. Rep.* **6**, 32277; doi: 10.1038/srep32277 (2016).

## Supplementary Material

Supplementary Information

## Figures and Tables

**Table 1:** Crystal and refinement data.

	Native	2 h	4 h	7 h	10 h
Wavelength (Å)	1.54	1.54	1.54	1.54	1.54
Resolution range (Å) (Last resolution shell)	21.86–1.60 (1.657–1.600)	30.73–1.61 (1.665–1.607)	27.95–1.61 (1.665–1.608)	27.84–1.61 (1.666–1.608)	33.33–1.61 (1.666–1.608)
Space group	P 4_3_ 2_1_ 2	P 4_3_ 2_1_ 2	P 4_3_ 2_1_ 2	P 4_3_ 2_1_ 2	P 4_3_ 2_1_ 2
Unit cell (Å)	78.82, 78.82, 36.82	78.84, 78.84, 36.84	79.04, 79.04, 36.6	78.75, 78.75, 36.94	78.65, 78.65, 36.80
Total reflections	31653 (3102)	29695 (2841)	30785 (2968)	29537 (2869)	27737 (2708)
Unique reflections	15835 (1551)	15398 (1471)	15566 (1499)	15205 (1489)	15261 (1458)
Multiplicity	2.0 (2.0)	1.9 (1.9)	2.0 (2.0)	1.9 (1.9)	1.8 (1.9)
Completeness (%)	99.92 (100.00)	98.50 (97.10)	99.79 (98.42)	97.40 (98.48)	98.19 (96.02)
Mean I/sigma(I)	53.22 (27.72)	14.29 (2.87)	30.22 (9.82)	21.95 (8.69)	11.62 (1.54)
Wilson B-factor (Å^2^)	10.68	16.38	14.74	11.59	16.54
R-merge (%)	0.0153 (0.0329)	0.0365 (0.2897)	0.0200 (0.0851)	0.0261 (0.0860)	0.0505 (0.4156)
R-meas (%)	0.0216	0.0516	0.0283	0.0370	0.0714
CC1/2	0.999 (0.996)	0.998 (0.736)	0.999 (0.971)	0.998 (0.972)	0.998 (0.606)
CC*	1 (0.999)	1 (0.921)	1 (0.993)	1 (0.993)	0.999 (0.869)
R-work	0.1711 (0.1614)	0.1776 (0.2294)	0.1645 (0.1786)	0.1697 (0.1672)	0.1816 (0.2710)
R-free	0.2106 (0.2336)	0.2127 (0.2382)	0.1993 (0.2580)	0.1870 (0.1699)	0.2110 (0.2999)
Number of non-hydrogen atoms	1206	1183	1197	1200	1223
Macromolecules	1008	1009	1001	1001	1001
Ligands	0	30	31	49	85
Water	198	144	165	150	137
Protein residues	129	129	129	129	129
RMS bonds (Å)	0.006	0.006	0.008	0.006	0.009
RMS angles (°)	1.05	1.01	1.04	1.01	1.09
Ramachandran favoured (%)	98	98	98	98	98
Ramachandran outliers (%)	0	0	0	0.79	0
Clash score	3.03	2.00	2.51	3.98	6.85
Average B-factor (Å^2^)	14.10	19.40	18.30	14.20	20.90
Macromolecules	12.10	17.90	16.50	12.40	19.00
Ligands	—	19.90	23.20	17.50	25.10
Solvent	23.90	29.90	27.70	25.10	32.00

**Table 2:** Protein-solvent (urea/water) hydrogen bonds (H-bonds).

Structure	Urea-MC H-bonds			Urea-SC H-bonds: Number, (% of total)	Urea – Protein H –bonds: Total (per urea)	Water – MC H-bonds			Water – SC H-bonds: Number, (% of total)	Water – Protein hydrogen bonds: Total (per water)	Total number of protein -solvent H- bonds
	Number, (% of total)	O_U_ –NH Number, (Av. Length (Å))	N_U_ – CO Number, (Av. Length (Å))			Number (% of total)	O_W_ – NH Number, (Av. Length (Å))	O_W_ – CO Number, (Av. Length (Å))			
Native						110 (53)	39 (3.0)	71 (2.8)	97(47)	207 (1.54)	207
2 h	13 (67)	3(2.8)	10 (3.0)	6(33)	19 (2.7)	81(54)	28 (3.0)	53(2.9)	68 (46)	149 (1.30)	168
4 h	12(75)	4 (3.0)	8 (3.1)	4 (25)	16 (2.3)	97 (54)	33(3.0)	64 (2.9)	83 (46)	180 (1.67)	196
7 h	17 (57)	6 (2.9)	11(3.0)	13(43)	30 (2.5)	82 (52)	24(3.0)	58(2.8)	77 (48)	159 (1.46)	189
10 h	23(44)	7 (2.9)	16 (3.0)	29 (56)	52 (2.5)	63 (52)	19 (3.1)	44 (2.8)	58 (48)	121 (1.48)	173

**Table 3:** Protein-solvent (urea/water) van der Waals (VW) contacts.

Structure	Number of First Shell waters	Water – Protein VW contacts					Number of First Shell urea	Urea-Protein VW contacts				
		Total (Backbone)	Backbone/total	Apolar	Polar	Polar/apolar		Total (Backbone)	Backbone/total	Apolar	Polar	Polar/apolar
Native	134	1539 (759)	0.49	615	924	1.56	—	—	—	—	—	—
2 h	114	1156 (601)	0.52	426	730	1.72	7	278 (155)	0.56	135	143	1.06
4 h	108	1338 (658)	0.49	504	834	1.67	7	268 (161)	0.60	125	143	1.15
7 h	109	1224 (608)	0.50	450	774	1.72	12	424 (196)	0.46	198	226	1.15
10 h	82	1028 (520)	0.51	393	635	1.61	21	600 (262)	0.44	277	323	1.16
