# Supplementary material for: Time-dependent X-ray diffraction studies on urea/hen egg white lysozyme complexes reveal structural changes that indicate onset of denaturation
Source: Sci Rep. 2016 Aug 30;6:32277. doi: 10.1038/srep32277 (PMC5004150; doi:10.1038/srep32277)
Supplement: Supplementary Information [file srep32277-s1.pdf]

Time-dependent X-ray diffraction studies on urea/hen egg white lysozyme complexes reveal structural changes that indicate onset of denaturation

Tushar Raskar, Sagar Khavnekar and Madhusoodan Hosur *

Tata Memorial Centre/Advanced Centre for Treatment Research and Education in Cancer, Kharghar, Navi Mumbai – 410210, India.

*email: mhosur@actrec.gov.in

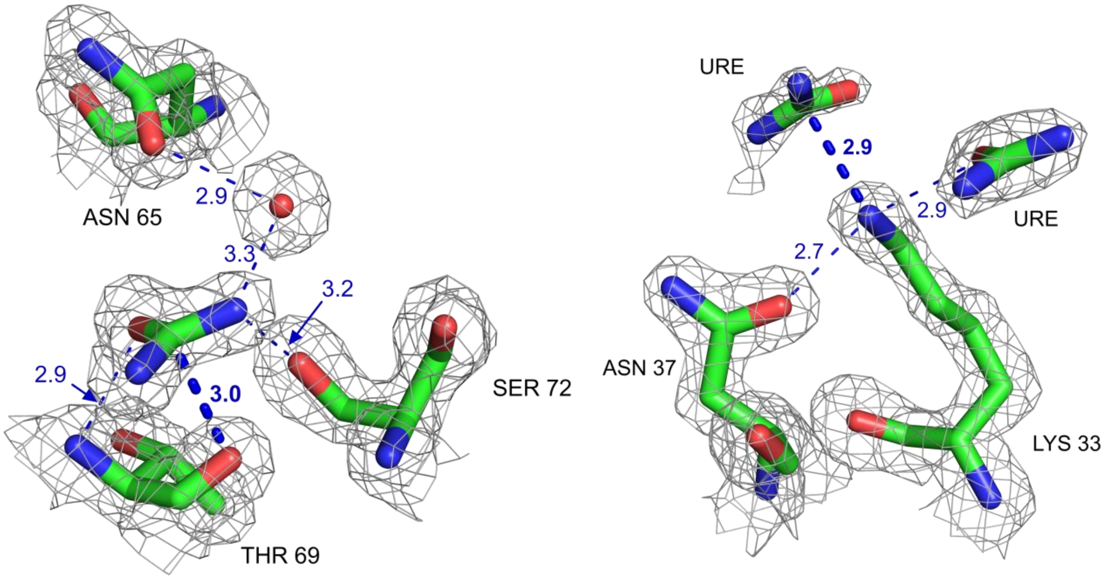

(a) (b)

Supplementary figure S1: Novel types of direct interactions between urea and lysozyme: a) H-bonds and stacking interactions with Thr 69, b) Lys 33 NH…Pi cloud from urea.

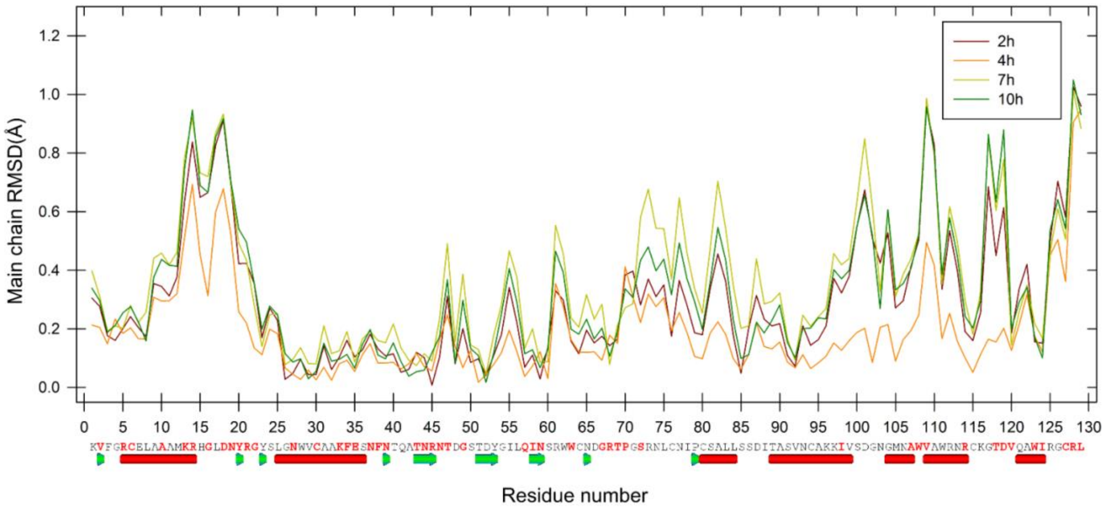

(a)

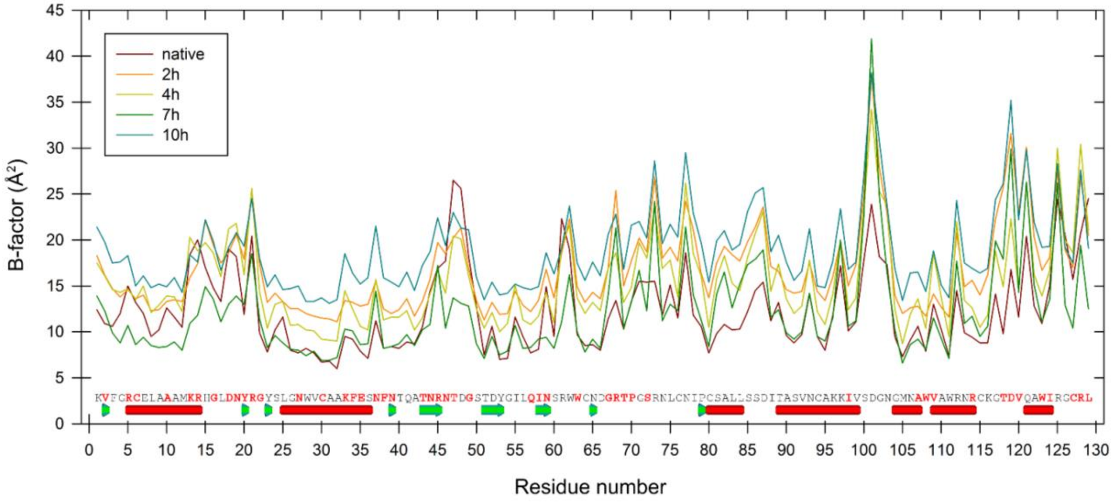

(b)

Supplementary Figure S2: (a) RMSD over 129 Cα's, compared to native, (b) B-factor comparisons for native, 2 h, 4 h, 7 h and 10 h. Secondary structure elements (helices as red cylinders and β-strands as green arrows) are also shown.

Supplementary Table S1: First shell water – water and water - urea hydrogen bonds.

Structure	Number First Shell Waters	Number of Water-water H-bonds	Average H-bond length (Å)	Number of urea molecules	Number of urea-water H-bonds	Average H-bond length (Å)	Hbnd2N (Å) (number)	Hbnd2O (Å) (number)	No. of water/No. of urea
Native	132	132	2.95	-	-	-	-	-	-
2 h	114	88	2.84	7	11	3.03	3.03(8)	3.04(3)	16.0
4 h	108	93	2.83	7	10	2.93	2.90 (6)	2.97(4)	15.4
7 h	109	92	2.84	12	19	2.91	2.94(13)	2.85(6)	9.0
10 h	83	58	2.75	21	31	2.90	2.91(19)	2.90(12)	3.9
